# Severe soft tissue injuries in multiple trauma patients—a challenge we can meet? A matched-pair analysis from the TraumaRegister DGU^®^

**DOI:** 10.3389/fmed.2025.1508172

**Published:** 2025-02-03

**Authors:** Nora Kirsten, Georg Maximilian Franke, Rolf Lefering, Tim Klüter, Matthias Weuster, Michael Müller, Sebastian Lippross, Andreas Seekamp, TraumaRegister DGU®, Stefanie Fitschen-Oestern

**Affiliations:** ^1^Department of Trauma Surgery, Hannover Medical School, Hannover, Germany; ^2^Department of Trauma Surgery, University Medical Center of Schleswig-Holstein, Kiel, Germany; ^3^Institute for Research in Operative Medicine (IFOM), University Witten/Herdecke, Cologne, Germany; ^4^Department of Trauma Surgery, DIAKO Hospital Flensburg, Flensburg, Germany; ^5^Department of Surgical Sciences, University of Otago, Dunedin, New Zealand

**Keywords:** severe soft tissue injuries, multiple trauma, TraumaRegister DGU^®^, sepsis, polytrauma care

## Abstract

**Introduction:**

Despite tremendous clinical efforts over the past few decades, the treatment of severely injured patients remains still challenging. Concomitant soft tissue injuries represent a particular challenge, as they can lead to complications at any time of trauma care, hold a high risk of infection and often require multiple surgical interventions and interdisciplinary collaboration.

**Methods:**

This retrospective, multicentric study used the TraumaRegister DGU^®^ to examine the effect of open fractures and severe soft tissue injuries on outcome of multiple trauma patients. Primary admitted multiple trauma patients at the age of 16 to 70 years, treated from 2010 to 2021, were included. A Matched pair analysis was performed for better comparability of trauma patients with and without open fractures and/or severe soft tissue injuries.

**Results:**

After applying the matching criteria, 5,795 pairs were created and analyzed. The group with sustained soft tissue injuries/open fractures was found to have a higher ISS ([mean ± SD] 22.1 ± 10.4 vs. 20.6 ± 10.2, *p* < 0.001). Endotracheal tube insertion (27.7% vs. 30.4%, *p* = 0.003), catecholamine administration (6.0% vs. 8.4%, *p* < 0.001) and cardio-pulmonary resuscitation (1.6% vs. 2.1%, *p* = 0.027) were more frequent in the group with sustained soft tissue injury. Both groups were equally frequent admitted to the intensive care unit (ICU) and length of stay (LOS) at the ICU (median (quartiles) 3 (1–9) versus 3 (1–9)) did not differ significantly. However, total LOS at the hospital was longer for the group with sustained soft tissue injury (median (quartiles) 18 (11–29) versus 17 (10–27)). Sepsis occurred more often in patients with soft tissue injury (4.3% vs. 5.2%, *p* = 0.034). There was no significant difference in prevalence of multi organ failure, 24 h-mortality (2.1% vs. 2.5%, *p* = 0.151) and overall-mortality (3.6% vs. 3.9%, *p* = 0.329) between both groups.

**Conclusion:**

Due to database analysis and revision of guidelines, the treatment of severely injured patients has steadily improved in recent years. Patients with severe soft tissue injuries/open fractures required more medical interventions and length of stay at the hospital was longer. In this study, we were able to show that although concomitant severe soft tissue injuries required more ICU interventions and led to a longer length of stay, 24-h and all-cause mortality were not significantly increased.

## 1 Introduction

Severe soft tissue injuries and open fractures remain a challenge in multiple trauma care. Open fractures are considered as traumatological emergencies even in isolated injuries (1). Severe soft tissue injuries result in tissue hypoxia, acidosis, and permeability damage to the vessels. In severely injured patients with general hypoxia, this effect is even exacerbated (2).

The outcome of trauma patients with open fractures and soft tissue injuries can vary depending on the severity of the injury, the extent of soft tissue damage, and the effectiveness of treatment. Age, preexisting conditions, gender, and prior medication may favor the occurrence of complications and negatively affect outcome (3, 4).

Major complication of patients with open fracture represents infection rate, which correlates with the severity of soft-tissue injury (5). Important long-term complications include osteitis and failure of bone healing.

The timing of wound management correlates with the risk of infection (5). Poor wound healing caused by infection can delay bone healing and lead to a longer hospital stay and prolonged recovery. Quality of resuscitation strategies and surgical concepts can minimize secondary complications (6).

The main principles of treatment include debridement and lavage, early antibiotic coverage, temporary or definitive stabilization of the skeletal injury and soft tissue coverage or reconstruction (7, 8).

Surgical treatment of extensive soft tissue injuries requires coordination between plastic surgery and trauma surgery (9). Essential improvements in multiple trauma care have occurred within the last decades, which have positive impact on management of severe soft tissue injuries.

With the following evaluation we wanted to investigate whether severe soft tissue injuries still lead to complications and worse outcome.

We used the TraumaRegister DGU^®^ to examine the effect of open fractures and severe soft tissue injuries on outcome of multiple trauma patients. Therefore, we evaluated comparable patients with open fractures or severe soft tissue injuries versus patients without open fractures and without severe soft tissue injuries.

## 2 Methods

### 2.1 TraumaRegister DGU^®^

The TraumaRegister DGU^®^ of the German Trauma Society (Deutsche Gesellschaft für Unfallchirurgie, DGU) was founded in 1993. The aim of this multi-centre database is a pseudonymized and standardized documentation of severely injured patients.

Data are collected prospectively in four consecutive time phases from the site of the accident until discharge from hospital: (A) Pre-hospital phase, (B) Emergency room and initial surgery, (C) Intensive care unit and (D) Discharge. The documentation includes detailed information on demographics, injury pattern, comorbidities, pre-and in-hospital management, course on intensive care unit, relevant laboratory findings including data on transfusion and outcome of each individual. The inclusion criterion is admission to hospital via emergency room with subsequent ICU/ICM care (Intensive Care Unit, Intermediate Care Unit) or reach the hospital with vital signs and die before admission to ICU. The infrastructure for documentation, data management, and data analysis is provided by AUC - Academy for Trauma Surgery (AUC - Akademie der Unfallchirurgie GmbH), a company affiliated to the German Trauma Society. The scientific leadership is provided by the Committee on Emergency Medicine, Intensive Care and Trauma Management (Sektion NIS) of the German Trauma Society. The participating hospitals submit their data pseudonymized into a central database via a web-based application. Scientific data analysis is approved according to a peer review procedure laid down in the publication guideline of TraumaRegister DGU^®^. The participating hospitals are primarily located in Germany (90%), but a rising number of hospitals of other countries contribute data as well. Participation in TraumaRegister DGU^®^ is voluntary. For hospitals associated with TraumaNetzwerk DGU^®^, however, the entry of at least a basic data set is obligatory for reasons of quality assurance.

This publication is in line with the publication guidelines of the TraumaRegister DGU^®^ and registered as TR-DGU project ID-2021-018.

### 2.2 Study cohort

For this study, primary admitted patients treated from 2010 to 2021 were included. To ensure comparability, only German-speaking countries were considered. Age range was set between 16 and 70 years. Cases with missing data, early transfer out, and patients with only minor injuries maximal abbreviated injury scale (MAIS) < 3 were excluded. Patients with severe head injury, degloving injury and penetrating trauma were excluded as we assumed that these injury patterns would influence mortality independently of soft tissue injury. Soft tissue injury was identified based on AIS scores (Abbreviated Injury Score). Abbreviated Injury Score is on a scale of one to six, one being a minor injury and six being fatal injury. There is a Skin/subcutaneous/muscle section in the AIS for each body region, which is categorized according to severity of 1, 2, and 3.

Minor soft tissue injuries without clinical relevance were not considered (AIS 1). Severe soft tissue injuries were defined as soft tissue injuries with an AIS of 2 or more.

The AIS codes for open fractures of the humerus (751272.3), radius (752272.3), ulna (752274.3), tibia (854001.3) and pelvis (856152.3, 856162.4) were included. The TraumaRegister DGU^®^ does not distinguish between open and closed femur fractures. Therefore, patients with femur fractures were also excluded. Complete data were available for 38,042 patients, of whom 7,200 had open fractures and severe soft tissue injury.

### 2.3 Definitions

To assess injury severity, the Abbreviated Injury Scale (AIS, Version 2005/Update 2008, Association for the Advancement of Automotive Medicine, Barrington) was applied. AIS is an injury severity scoring system, which classifies each injury by body region on a six-point scale, ranging from 1 = minor to 6 = maximal. Based on AIS, the injury severity score (ISS) is calculated to provide a numerical description of the overall severity of injury in multiple trauma patients (10). Sepsis was defined based on the sepsis-3 definition (11). Multiple organ failure (MOF) was assessed using the sequential organ failure assessment (SOFA) (12), whereas two or more systems with SOFA >2 were required to diagnose MOF.

As outcome parameters, we examined mortality, length of stay in the intensive care unit and in the hospital and complications.

### 2.4 Statistical analyses

Statistical analyses were performed with SPSS 24.0. (IBM, Armonk, NY, United States). Continuous and categorical variables are presented as mean ± standard deviation (SD) or as quartiles in case of rather skewed measurements. Categorical variables are presented as numbers (percentages) respectively. To evaluate the independent impact of soft tissue injury in severely injured patients, a matched pair analysis was performed. The matching criteria are listed in Table 1. Comparable trauma patients with or without soft tissue injury were then paired, resulting in 5795 pairs. Data between groups were compared using Pearson’s chi-squared test for categorial variables and two-tailed t-test for continuous variables. Values were considered statistically significant when *p* < 0.05. The Revised Injury Severity Classification version II (RISC-II) was applied to predict mortality. Outcome adjustment was calculated based on worst and second-worst injury (AIS severity level), head injury, age, sex, pupil reactivity and size, pre-injury health status, blood pressure, acidosis (base deficit), coagulation, hemoglobin, and cardiopulmonary resuscitation (13).

**Table 1:** The following matching listed criteria were used to match 5,795 pairs of patients with severe soft tissue injuries/open fractures and patients without severe soft tissue injuries/open fractures.

Matching criteria	Specification/grouping
Age group (years)	16–39 / 40–54 / 55–70
Sex	Male / female
ASA score before injury	1–2 / 3–4
Concomitant head injury (AIS)	0–1 / 2–3
Concomitant arm injury (AIS)	0–1 / 2–3
Concomitant leg injury (AIS)	0–1 / 2–3
Concomitant thoracic injury (AIS)	0–1 / 2–3 / 4–5
Concomitant abdominal injury (AIS)	0–1 / 2–3 / 4–5
Concomitant pelvic injury (AIS)	0–1 / 2 / >3

ASA, American Society of Anesthesiologists; AIS, Abbreviated injury scale.

## 3 Results

**Figure 1:**
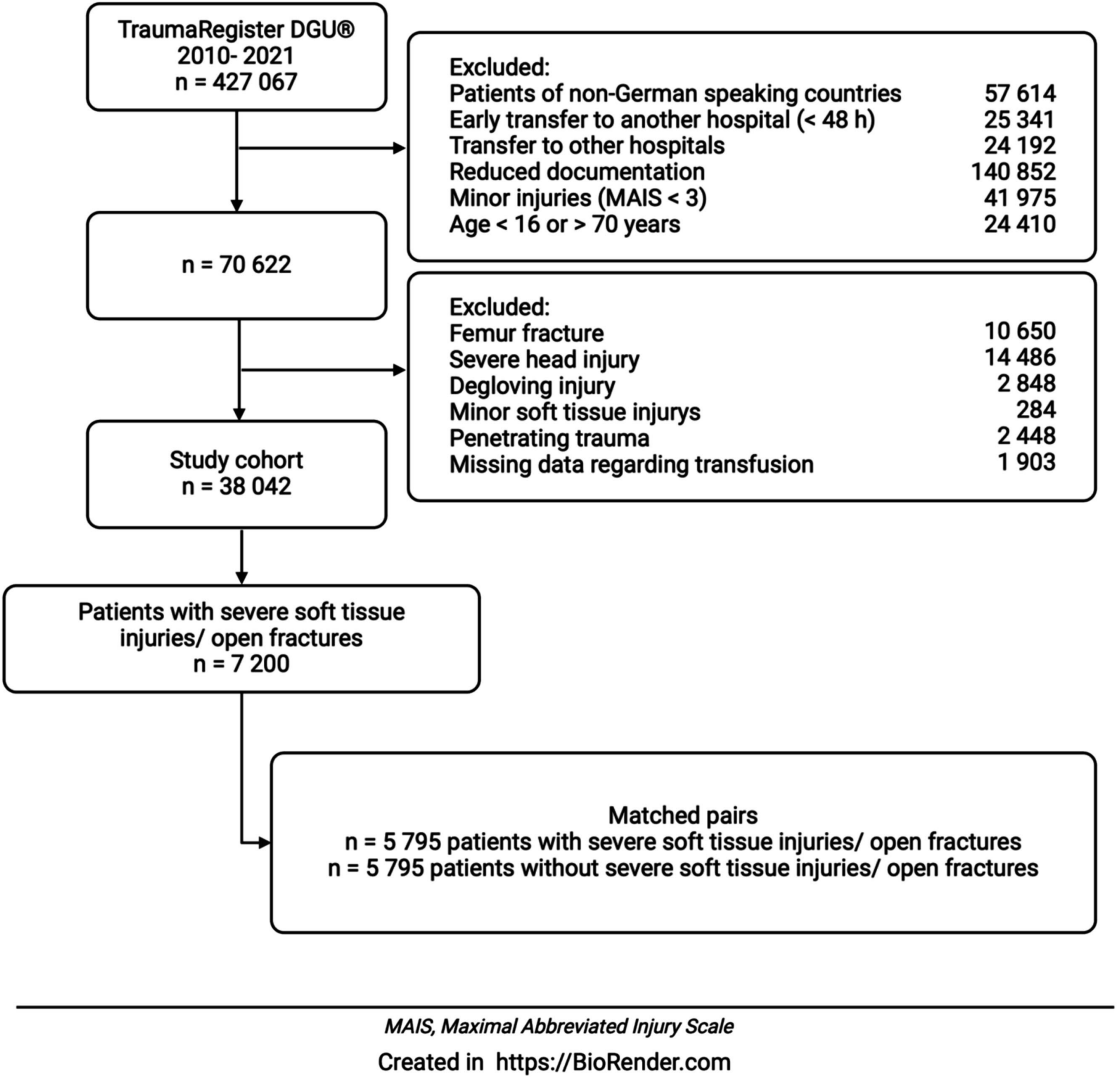
After applying the exclusion criteria, 38,042 patients were included in the evaluation. A severe soft tissue injury was diagnosed in 7200 patients. In a matched pair analysis, 5,795 patients with a severe soft tissue injury or open fracture were compared with 5,795 patients without a severe soft tissue injury.

A total of 38,042 patients met the inclusion criteria (Figure 1). After matching based on the criteria listed in Table 1, 5,795 pairs were created and analyzed for the impact of severe soft tissue injury on the outcome of severely injured patients.

### 3.1 Baseline characteristics

For 38,042 patients found eligible after applying above mentioned selection criteria (see Figure 1), baseline data are shown in Table 2. Patients with soft tissue injury were younger than the control group. Male gender was evenly distributed. Patients without sustained soft tissue injury tended to have a higher ASA score, although the difference was not statistically significant. Mean ISS was higher in patients with soft tissue injury. Additionally, more diagnoses were assigned for the group with soft tissue injury. The majorities of patients in both groups were admitted to level 1 trauma centers. However, for patients with soft tissue injury, treatment was carried out more often in level 1 trauma centers when compared to the control group.

**Table 2:** Distribution of baseline criteria of patients with severe soft tissue injuries and open fractures and without severe soft tissue injuries/open fractures (*n* = 38,042).

	Control (*n* = 30,842)	Soft tissue injury /open fracture (*n* = 7,200)	*p* value
Age, years, mean ± SD	45.3 ± 15.32	43.8 ± 15.1	< 0.001
16–39 years, n (%)	10,763 (34.9%)	2,757 (38.3%)	
40–54 years, n (%)	9,843 (31.9%)	2,375 (33.0%)	
55–70 years, n (%)	10,236 (33.2%)	2,068 (28.7%)	
Male sex, n (%)	23,204 (75.3%)	5,462 (76.0%)	0.242
ASA ≥ 3, n (%)	2,255 (7.3%)	482 (6.7%)	0.068
ISS, mean ± SD	18.8 ± 9.6	21.5 ± 11.0	< 0.001
Diagnoses, n, mean ± SD	4.6 ± 2.5	5.7 ± 3.2	< 0.001
Trauma center level of care			
Level 1, n (%)	25,213 (81.7%)	6,130 (85.1%)	
Level 2, n (%)	4,587 (14.9%)	870 (12.1%)	
Level 3, n (%)	1,042 (3.4%)	200 (2.8%)	

Statistical significance was calculated using two-sided t-test for nominal variables and chi-square-test for categorial variables, respectively. ASA, American Society of Anesthesiologists; ISS, Injury severity score.

### 3.2 Mode of accident

Table 3 presents the distribution of accident types for patients with and without severe soft tissue injury. Car accidents were the most common cause of accident in both groups. Motorbike, pedestrian and high falls were accident types, that more frequently were associated with soft tissue injuries. In contrast, bicycle and low falls were those more likely to have no soft tissue injury.

**Table 3:** Cause of accident of patients with severe soft tissue injuries and open fractures (*n* = 7,167) versus patients without severe soft tissue injuries/open fractures (*n* = 30,664).

	Control *n* = 30,664	Soft tissue injury/ open fracture *n* = 7,164
Car, n (%)	7,349 (24.0%)	1,778 (24.8%)
Motorbike, n (%)	5,333 (17.4%)	1,621 (22.6%)
Bicycle, n (%)	3,431 (11.2%)	511 (7.1%)
Pedestrian, n (%)	1,404 (4.6%)	621 (8.7%)
High fall, n (%)	5,841 (19.1%)	1,417 (19.8%)
Low fall, n (%)	4,402 (14.4%)	480 (6.7%)
Others, n (%)	2,904 (9.5%)	736 (10.3%)

### 3.3 Injury pattern

The distribution of the injuries of patients with and without severe soft tissue injury was analyzed and displayed in Table 4. Severe soft tissue injury was more frequent on patients with injuries of the upper or lower extremities. Thorax or spinal trauma was more often associated with closed injuries.

**Table 4:** Distribution of severe injuries in patients without soft tissue injury versus with soft tissue injury/open fracture (*n* = 38,042).

	Control (*n* = 30,842)	Soft tissue injury/ open fracture (*n* = 7,200)	*p* value
Concomitant head injury AIS ≥ 2, n (%)	10,822 (35.1%)	2,368 (32.9%)	<0.001
Concomitant thoracic injury AIS ≥ 2, n (%)	20,809 (67.5%)	3,751 (52.1%)	<0.001
Concomitant abdominal injury AIS ≥ 2, n (%)	6,312 (20.5%)	1,547 (21.5%)	0.054
Concomitant spine injury AIS ≥ 2, n (%)	12,386 (40.2%)	2,484 (34.5%)	<0.001
Concomitant arm injury AIS ≥ 2, n (%)	9,961 (32.3%)	3,752 (52.0%)	<0.001
Concomitant pelvic or leg injury AIS ≥ 2, n (%)	4,285 (13.9%)	3,765 (52.3%)	<0.001

AIS, Abbreviated injury scale.

### 3.4 Baseline characteristics after matched-pair analysis

After we first analyzed the whole study population, a matched pair analysis was carried out. The baseline characteristics were assessed and displayed in Table 5. Applying the age groups listed in Table 1, the age was equally distributed between both groups. The group with sustained soft tissue injury had a higher ISS and more listed diagnoses than the control group. For both groups, the vast majority was treated in level 1 trauma centers. Approximately 12% were admitted to level 2, and 2.8% or 2.6%, respectively, to level 3 trauma centers.

**Table 5:** Baseline characteristics of patients with and without soft tissue injury/open fracture after matched-pair analysis.

	Control (*n* = 5,795)	Soft tissue injury/ open fracture (*n* = 5,795)	*p* value
Age, years, mean ± SD	43.7 ± 15.3	43.8 ± 15.1	0.62
ISS, mean ± SD	20.6 ± 10.2	22.1 ± 10.4	< 0.001
Diagnoses, n, mean ± SD	5.3 ± 2.7	5.8 ± 3.1	< 0.001
Trauma center level of care			
Supraregional, n (%)	4,943 (85.3%)	4,960 (85.4%)	
Regional, n (%)	689 (11.9%)	696 (12%)	
Local, n (%)	163 (2.8%)	139 (2.6%)	

5,795 patients with a severe soft tissue injury or open fracture were compared with 5,795 patients without a severe soft tissue injury. Statistical significance was calculated using two-tailed *t*-test.

### 3.5 Mode of accident after matched-pair analysis

A comparison of accident type showed a higher proportion of car accidents (23.9% vs. 26.4%) and pedestrian accidents (7.0% vs. 8.3%) for the group with soft tissue injury (Table 6). The incidences of motorbike accidents were the same. High and low fall as well as bicycle accident occurred more frequently the control group.

**Table 6:** Cause of accident of patients with severe soft tissue injuries/open fractures (5,795 patients) versus patients without severe soft tissue injuries/open fractures (5,795 patients) as part of the matched-pair analysis.

	Control (*n* = 5,773)	Soft tissue injury/ open fracture (*n* = 5,764)
Car, n (%)	1,380 (23.9%)	1,522 (26.4%)
Motorbike, n (%)	1,231 (21.3%)	1,221 (21.2%)
Bicycle, n (%)	445 (7.7%)	414 (7.2%)
Pedestrian, n (%)	402 (7.0%)	479 (8.3%)
High fall, n (%)	1,349 (23.4%)	1,163 (20.2%)
Low fall, n (%)	476 (8.2%)	374 (6.5%)
Others, n (%)	490 (8.5%)	591 (10.3%)

### 3.6 Treatment and outcome

Prehospital and inhospital treatment, complications, and outcome were analyzed and are shown in Table 7. Endotracheal tube insertion (27.7% vs. 30.4%, *p* = 0.003), catecholamine administration (6.0% vs. 8.4%, *p* < 0.001) and cardio-pulmonary resuscitation (CPR) (1.6% vs. 2.1%, *p* = 0.027) were more frequent in the group with sustained soft tissue injury. In addition, patients with sustained soft tissue injury received more intravenous (i.v.) fluids ([mean ± SD, mL] 835.8 ± 638.4 vs. 885.94 ± 667, *p* < 0.001) and administration of packed red blood cells (pRBC) (10.8% vs. 13.8%, *p* < 0.001) was more frequent when compared to the control group. The need for surgery (77.2% vs. 88.5%, *p* < 0.001) and the number of surgeries performed ([mean] ± SD, [n] 3.0 ± 3.5 vs. 4.6 ± 5.2, *p* < 0.001) was higher in the group with sustained soft tissue injury. Length of stay (LOS) at the ICU did not differ significantly between both groups. However, total LOS at the hospital was longer for the group with sustained soft tissue injury ([mean] (quantiles) 18 (11–29) versus 17 (10–27)). Sepsis occurred more often in patients with soft tissue injury (4.3% vs. 5.2%, *p* = 0.034). MOF also tended to occur more frequent in the group with sustained soft tissue injury, but there was no significance. Mortality within first 24 h (2.1% vs. 2.5%, *p* = 0.151) and overall-mortality (3.6% vs. 3.9%, *p* = 0.329) did not differ significantly between both groups. In both groups, expected mortality (RISC II score) exceeded observed overall-mortality.

**Table 7:** Study outcome of patients without soft tissue injury versus with soft tissue injure after matching (*n* = 11,590).

	Control (*n* = 5,795)	Soft tissue injury/ open fracture (*n* = 5,795)	*p*-value
Prehospital treatment			
Endotracheal tube, n (%)	1,584 (27.7%)	1728 (30.4%)	0.003
Catecholamines, n (%)	339 (6.0%)	479 (8.4%)	< 0.001
CPR, n (%)	89 (1.6%)	121 (2.1%)	0.027
I.v. fluids, mL, mean ± SD	835.8 ± 638.4	885.94 ± 667	< 0.001
Inhospital treatment			
pRBCs, n (%)	625 (10.8%)	797 (13.8%)	< 0.001
Surgery, n (%)	4,475 (77.2%)	5,131 (88.5%)	< 0.001
Number of surgeries, n, mean ± SD	3.0 ± 3.5	4.6 ± 5.2	< 0.001
ICU admission, n (%)	5,283 (91.2%)	5,235 (90.3%)	0.123
ICU LOS, d, mean ± SD	7.1 ± 12.5	7.2 ± 11.4	0.584
Hospital LOS, d, mean ± SD	21.6 ± 19.1	23.6 ± 21.5	< 0.001
Complications			
Sepsis, n (%)	221 (4.3%)	267 (5.2%)	0.034
MOF, n (%)	749 (14.3%)	808 (15.5%)	0.081
24 h-mortality, (n) %	120 (2.1%)	143 (2.5%)	0.151
Overall-mortality, (n) %	208 (3.6%)	228 (3.9%)	0.329
RISC II score, mean	4.0	4.5	0.059

Significances were calculated with two-sided *t*-test for nominal variables and chi-square-test for categorial variables, respectively. CPR, cardio-pulmonary resuscitation; i.v., intravenous; pRBC, packed red blood cells; ICU, intensive care unit; LOS, lenght of stay; MOF, multiple organ failure; RISC, RISC of death based on RISCII.

## 4 Discussion

Despite tremendous clinical efforts and advances in treatment over the past few decades, the treatment of severely injured patients remains challenging (14, 15). Severe soft tissue injuries and open fractures pose a particular challenge, as they can lead to complications at any time during multiple trauma care. These complications can be manifold including infection, osteomyelitis, posttraumatic osteoarthritis, and nonunion (16). Infection and severity of injury cause longer hospital stay and higher mortality rate (17).

Both, mechanical and ischaemic tissue damage play a decisive role in trauma management (16). Posttraumatic inflammatory response can be activated by bacteria that enter via open injuries or by release of inflammatory markers via tissue damage (2). Immune modulation predisposes trauma patients to higher infection rate, sepsis and multi-organ dysfunction (2).

The purpose of this study was to investigate the epidemiology and following, the impact of severe soft tissue injury and open fracture as an independent variable on the outcome of multiple trauma patients.

In general, trauma patients with sustained soft tissue injury were significantly younger and had a higher ISS compared to patients without soft tissue injuries. Severe soft tissue injuries in younger trauma patients are mainly caused by high energy trauma related to traffic accidents (18). An elderly peak is primary caused by fall from standing (19). Extended soft tissue damage with active bleeding causes higher AIS and higher Injury Severity Score.

Since many systemic complications have been described to occur in patients with open fractures, they are more likely to be treated in a level 1 trauma center (20). Further, irrespective to zone of injury, management of open fractures involves temporary and definitive stabilization, debridement and final soft tissue coverage (21). Number of operations and diagnosis was higher in trauma patients with severe soft tissue injuries which was accompanied by higher RISC II and a longer hospital stay. Higher RISC II correlates with higher ISS in this group.

In multiple trauma patients, early flap plastic is not possible due to severity of injuries, tissue swelling and soiling of the wound. Negative pressure wound therapy (NPWT) has established itself as the primary therapeutic tool for severely injured patients with extensive soft tissue defects. If primary wound closure is not possible due to the risk of infection or the size of the defect, the use of NPWT is recommended in various guidelines worldwide (22, 23). Surgical debridement play an important role in preventing the risk of infection after extended soft tissue injuries (24). The timing of surgical flap plastic can be discussed controversial (25), but early surgical debridement have been shown to reduce risk of infection and sepsis (24). Otherwise, numerous studies show that tissue damage due to surgery or trauma increases the patient’s susceptibility to infection (2). By pre-activating the cell trough damage-associated molecular patterns, tissue damage can amplify the response of the innate immune system and increase inflammation (26). Kobbe et al. (27) showed, that both fracture and soft tissue injury can trigger a systemic inflammatory response. But the combination of fracture and STI can even lead to a marked liver disfunction (27). Fan and colleagues showed that hemorrhagic shock promotes acute lung injury trough upregulated TLR4 signaling (28). These results suggest that severe soft tissue injury can lead to sepsis or multiple organ failure.

According to matched-pair analysis, we found that the incidence of sepsis was higher in the group with severe soft tissue injury/open fracture, but there was no significant difference in the incidence of MOF. Compared to previous studies, the proportion of patients with sepsis has been reduced over the last decades (29). Irrespective of open injuries, various independent predictors have been identified that favor sepsis (29). The possibility to recognize predictors and initiate early antibiotic treatment is considered as an essential step to reduce septic shock and mortality in trauma patients (30). Knowledge of posttraumatic inflammatory reaction and rapid treatment of open fractures has made a decisive contribution to reducing complications in general.

Matched-pair analysis showed that trauma patients with severe soft tissue injuries required more often intubation and administration of catecholamines. Increased blood loss caused higher fluid replacement and blood substitution. Long bones are highly vascular, and fracture can result in significant bleeding (31). Furthermore, in the case of an open fracture, there is no closed compartment so that blood loss is not limited and can double compared to closed fractures (32). Higher fluid supply, higher intubation rate and significant more reanimations in patients with soft tissue injuries and open fractures can be attributed to the four interactive cycles coagulopathy, hypothermia, hemorrhage and tissue injury, which interact with each other (2, 37).

Number of surgical interventions was significantly higher in patients with soft tissue damage. Several debridement and application of external fixator are often required before final wound closure or flap coverage is possible. Timing of radical debridement and antibiotic prophylaxis can reduce the risk of complications after severe soft tissue injuries significantly (33).

Injury severity has an impact on complication rate and mortality after multiple trauma (34, 35). Although there was a higher risk of various complications in patients with severe soft tissue injuries, mortality rate within 24 h and overall mortality were not affected. Comparable mortality rates indicate a significant improvement in trauma management and revised treatment of complications. Yoo et al. (36) focused on open versus closed pelvic fracture and found similar results. Early debridement, wound management and proper fracture fixation have a significant impact on clinical course and outcome (24).

Trauma care and management of severe soft tissue injuries and open fractures have improved in recent years to an extent that open fractures and severe soft tissue injuries are not a predictor of increased mortality furthermore.

## 5 Conclusion

Multiple trauma patients with severe soft tissue injuries and open fractures tend to be younger and were more seriously injured compared to patients without severe soft tissue injuries. Severe soft tissue injuries are essentially caused by traffic accidents and high energy trauma.

Matched-pair analysis showed significantly higher fluid and blood administration, donation of catecholamines and intubations.

Patients with severe soft tissue injuries had significantly more diagnoses and more surgical interventions. Although sepsis was more frequently detected as a complication, we could not find a significant difference in MOF, mortality rate within 24 h and total mortality.

The experience of trauma management, correct assessment, early debridement and fracture stabilization may have contributed to this significant reduction in complications and overall mortality following open soft tissue injuries.

## 6 Limitations

This is a retrospective analysis of data provided by the TraumaRegister DGU^®^. Data of 38,042 patients were evaluated. Even if the data quality is very high, it should be recognized that in large registries, some data sets might not be complete.

Minor soft tissue injuries rarely lead to complications. We focused only on severe soft tissue injuries and open fractures in multiple trauma patients. Degloving injuries were excluded because these injuries can be open or closed and the TraumaRegister DGU^®^ does not differentiate between open and closed decollement injuries. The extent of the decollement or degloving injuries often only becomes apparent during the further course of trauma care. Multiple trauma patients who died before hospitalization were not included. Patients who were transferred after admission could not be included due to missing data. Only the data of patients up to discharge from the primary treating hospital were evaluated.

Further, the included patients were treated by different emergency physicians and emergency teams, whose level of training and experience in emergency care were not considered.

The presented study demonstrates that polytrauma patients with soft tissue injuries do not have significantly increased mortality. Future studies could focus on identifying treatment parameters associated with improved outcomes, such as antibiotic administration or NPWT. However, these parameters are not documented in the TraumaRegister DGU^®^ and require further investigation. This study only analyzes data from German-speaking countries. Further international studies are required for a broader comparison.

Another limitation of the study is the evaluation of complications. Long-term complications such as osteitis and pseudarthrosis in the absence of fracture healing are not documented in the TraumaRegister DGU^®^.

## Data Availability

The original contributions presented in the study are included in the article/supplementary material, further inquiries can be directed to the corresponding author.

## Group member of TraumaRegister DGU^®^

Committee on Emergency Medicine, Intensive Care and Trauma Management (Sektion NIS) of the German Trauma Society (DGU), Germany.
